# Differences between early versus late correction of Tetralogy of Fallot (TOF) in cardiac Magnetic Resonance (CMR)

**DOI:** 10.1186/1532-429X-13-S1-P193

**Published:** 2011-02-02

**Authors:** Matthias Grothoff, Janine Hoffmann, Kai Boegershausen, Matthias Gutberlet

**Affiliations:** 1 Leipzig Heart Center, Leipzig, Germany

## Introduction

Tetralogy of Fallot can be repaired with low mortality and most patients reach adulthood. Nevertheless the optimal timing of surgery remains controversial.

## Purpose

To evaluate differences between early versus late correction of Tetralogy of Fallot (TOF) in cardiac Magnetic Resonance (CMR).

## Methods

CMR was performed in 55 patients (20 male) using a 1.5T scanner. RV-volumes and pulmonary-regurgitant-fractions (PRF) were calculated from standard cine-sequences and flow-sensitive gradient-echo images, respectively. Scar tissue was quantified from Delayed Enhancement (DE) imaging. Patients were divided into two groups depending age at total repair (group 1≤1year, n=25; group2>1years, n=30).

## Results

In 50 patients (91%) RV image quality was diagnostic for quantification of RV DE. Patients of group 2 demonstrated with a significantly higher RV DE (p<0.05) compared to group 1. No differences were found in regard to LV DE, RV size and RV function.

## Conclusions

Patients repaired <1 year show a lower amount of fibrous tissue in the RV. This might support the strategy of early repair.

